# Curcumin-activated Olfactory Ensheathing Cells Improve Functional Recovery After Spinal Cord Injury by Modulating Microglia Polarization Through APOE/TREM2/NF-κB Signaling Pathway

**DOI:** 10.1007/s11481-023-10081-y

**Published:** 2023-09-02

**Authors:** Chao Jiang, Zhe Chen, Xiaohui Wang, Yongyuan Zhang, Xinyu Guo, Hong Fan, Dageng Huang, Yuqing He, Xiangwen Tang, Yixiang Ai, Youjun Liu, Hao Yang, Dingjun Hao

**Affiliations:** 1 Department of Spine Surgery, Hong Hui Hospital, Xi’an Jiaotong University, Xi’an, 710054 China; 2 Shaanxi Key Laboratory of Spine Bionic Treatment, Xi’an, 710054 China; 3 Translational Medicine Center, Hong Hui Hospital, Xi’an Jiaotong University, Xi’an, 710054 China; 4 Department of Neurology, The Second Affiliated Hospital of Xi’an Jiaotong University, Xi’an, 710004 China; 5 Basic Medical School Academy, Shaanxi University of Traditional Chinese Medicine, Xianyang, 712046 China; 6 Department of spine Surgery, Hong Hui Hospital, Xi’an Jiaotong University, Shaanxi Key Laboratory of Spine Bionic Treatment, Xi’an, 710054 China

**Keywords:** Curcumin, Olfactory ensheathing cells, Spinal cord injury, Microglia polarization, Apolipoprotein E, Triggering receptor expressed on myeloid cells 2

## Abstract

Transplantation of curcumin-activated olfactory ensheathing cells (aOECs) improved functional recovery in spinal cord injury (SCI) rats. Nevertheless, little is known considering the underlying mechanisms. At the present study, we investigated the promotion of regeneration and functional recovery after transplantation of aOECs into rats with SCI and the possible underlying molecular mechanisms. Primary OECs were prepared from the olfactory bulb of rats, followed by treatment with 1µM CCM at 7–10 days of culture, resulting in cell activation. Concomitantly, rat SCI model was developed to evaluate the effects of transplantation of aOECs in vivo. Subsequently, microglia were isolated, stimulated with 100 ng/mL lipopolysaccharide (LPS) for 24 h to polarize to M1 phenotype and treated by aOECs conditional medium (aOECs-CM) and OECs conditional medium (OECs-CM), respectively. Changes in the expression of pro-inflammatory and anti-inflammatory phenotypic markers expression were detected using western blotting and immunofluorescence staining, respectively. Finally, a series of molecular biological experiments including knock-down of triggering receptor expressed on myeloid cells 2 (TREM2) and analysis of the level of apolipoprotein E (APOE) expression were performed to investigate the underlying mechanism of involvement of CCM-activated OECs in modulating microglia polarization, leading to neural regeneration and function recovery. CCM-activated OECs effectively attenuated deleterious inflammation by regulating microglia polarization from the pro-inflammatory (M1) to anti-inflammatory (M2) phenotype in SCI rats and facilitated functional recovery after SCI. In addition, microglial polarization to M2 elicited by aOECs-CM in LPS-induced microglia was effectively reversed when TREM2 expression was downregulated. More importantly, the in vitro findings indicated that aOECs-CM potentiating LPS-induced microglial polarization to M2 was partially mediated by the TREM2/nuclear factor kappa beta (NF-κB) signaling pathway. Besides, the expression of APOE significantly increased in CCM-treated OECs. CCM-activated OECs could alleviate inflammation after SCI by switching microglial polarization from M1 to M2, which was likely mediated by the APOE/TREM2/NF-κB pathway, and thus ameliorated neurological function. Therefore, the present finding is of paramount significance to enrich the understanding of underlying molecular mechanism of aOECs-based therapy and provide a novel therapeutic approach for treatment of SCI.

**Supplementary Information:**

The online version contains supplementary material available at 10.1007/s11481-023-10081-y.

## Background

Spinal cord injury (SCI) often occurs as a wide range of different insults to the normal anatomical structures and physiological functions of the spinal cord, which mainly results in a series of disastrous consequences such as irreversible motor deficits and sensory dysfunction (Sofroniew 2018; Yang et al. 2020). More importantly, neural repair is extremely difficult due to both intrinsic properties of neurons and formation of an extrinsic hostile environment (inflammation, ischemia, hypoxia and glial scars, etc.) after SCI (Mahar and Cavalli 2018; Afshari et al. 2009). With regard to the pathological and physiological features, SCI pathophysiology is usually categorized into primary and secondary injury phases (Hellenbrand et al. 2021). Primary damage is defined as the initial traumatic insult which is characterized by hemorrhage, edema, ischemia and hypoxia (Hellenbrand et al. 2021), followed by a progressive secondary injury characterized by inflammatory cascade, neural degeneration and scar formation (Oyinbo 2011). Among all the mechanisms of secondary damage, inflammatory responses, as the principal culprits in initiating the second phase, can greatly contribute to the severity of SCI. Therefore, developing the feasible and effective strategies to regulate the complicated pathophysiological processes and improve the deteriorating microenvironment within SCI is highly necessary. Cellular therapy can be an effective option to ameliorate SCI pathologies by modulating inflammation, inhibiting scar formation, and promoting axonal regeneration and angiogenesis (Assinck et al. 2017; Zhang et al. 2017; Gómez et al. 2018; López-Vales et al. 2004). Up to now, cells-based therapy has displayed excellent potential in treating SCI and has been attempted in preclinical studies.

At the present, multitudinous kinds of cells, mainly involving stem cells and non-stem cells, have been used for treating SCI (Assinck et al. 2017). Although cellular interventions have obtained favorable outcomes, there were some remarkable differences among different types of cells, mainly including olfactory ensheathing cells (OECs), Schwann cells (SCs), mesenchymal stem cells (MSCs) (Cofano et al. 2019; Ahuja et al. 2020; Ribeiro et al. 2023). In comparison with these cells, OECs have increasingly attracted more and more attention owing to their distinctive biological properties (Boyd et al. 2005; Yang et al. 2015). OECs, a unique type of glial cells derived from the olfactory system, play a pivotal role in the turnover of olfactory neurons throughout life-span (Boyd et al. 2005). Compelling studies have evidenced that OECs share excellent biological functions, such as phagocytic activity, neurite-promoting guidance and myelination capacity, which confer neuroprotection and promote neuronal regeneration and plasticity (Yang et al. 2015). In addition, OECs have also been demonstrated to exert substantially advantageous effects on SCI by modulating neuroinflammation, supporting neural regeneration, and promoting angiogenesis and neuronal survival (Ursavas et al. 2021; Jiang et al. 2022a, b; Wang et al. 2022). Accordingly, OECs have been considered as one of the most promising candidates for treatment of SCI. Nevertheless, the pro-regenerative potential of OECs still relies on several factors including cell viability, purity and source (Reshamwala et al. 2020). Therefore, potentiating the bio-functions of OECs is a prerequisite for achieving desirable outcomes. Curcumin (CCM), a natural polyphenol extracted from turmeric, has been demonstrated to have multiple bio-functions including anti-inflammation, anti-oxidation and neuroprotection which are propitious in mitigating neural injury (Strimpakos and Sharma 2008). Our previous studies also indicated that CCM and/or lipopolysaccharide (LPS) could activate OECs and then remarkably enhance their phagocytic capacity (Hao et al. 2017). In addition, CCM-activated OECs can improve neurological function after SCI by modulating the secretion of neurotrophins and inflammatory factors (Guo et al. 2020). Likewise, our latest study revealed that activated OECs promoted angiogenesis and improved microenvironment following SCI through PI3K/Akt pathway (Wang et al. 2022). Nonetheless, the exact mechanisms through which transplanted aOECs inhibit deleterious inflammation, leading to neural regeneration, is elusive.

It is well known that inflammatory cascade accompanying SCI is extremely detrimental to the recovery of neurological function (Anwar et al. 2016; David and Kroner 2011). Microglia, as the resident innate immune cells in central nervous system (CNS), are proposed as the predominant contributors to neuroinflammation. The prevalent notion holds that microglia can be classified as M1 and M2 phenotype (David and Kroner 2011). Specifically, M1 microglia expressed cell surface markers (iNOS, CD86 and CD16, etc.) and produced pro-inflammation cytokines (TNFα, IL-6, and IL-1β, etc.), while M2 subgroup expressed surface markers (Arg-1, CD206 and CD204, etc.) and anti-inflammation cytokines (TGF-β, IL-10 and BDNF, etc.) (David and Kroner 2011; Kigerl et al. 2009). Nevertheless, the switch of M1 to M2 is a dynamic process and it is hard to distinguish at certain stage. After SCI, M1 subgroup is predominant and has negative influence on neural survival and axonal extension by releasing pro-inflammation cytokines and other detrimental substances, whereas M2 subgroup has anti-inflammation and neuroprotective roles by producing anti-inflammatory factors and neurotrophins. Therefore, the effective polarization of M1 to M2 microglia may be a prospective clinical strategy for treatment SCI. Moreover, previous studies evidenced that the expression of M1 markers (iNOS and CD86) increased and M2 makers (Arg-1 and CD206) decreased in microglia treated by LPS and (or IFN-γ) (Zhai et al. 2017; Fan et al. 2019, 2022). Based on these findings, we explored the expression level of relevant markers including iNOS, CD86, Arg-1 and CD206 to identify the anti-inflammatory property of aOECs in vivo and in vitro. Although transplantation of OECs ameliorates inflammatory response and promotes functional recovery after SCI (Jiang et al. 2022a, b; Guo et al. 2020; Zhang et al. 2021), the underlying mechanisms have not been thoroughly elucidated. Triggering receptor expressed on myeloid cells 2 (TREM2), a member of the TREM family, is specifically expressed in microglia in the CNS and plays crucial roles in the modulation of inflammation (Zhai et al. 2017; Ulrich and Holtzman 2016; Sanjay, Shin et al. 2022). Recent literatures revealed that TREM2 could participate in regulating inflammation by microglial polarization from M1 to M2 (Zhai et al. 2017; Sanjay, Shin et al. 2022). However, whether OECs activated by CCM could participate in polarizing microglia and thus inhibit inflammation by targeting TREM2 is not known.

In the current study, we firstly explored the effects of transplantation of aOECs on neural regeneration in rats after SCI and possible mechanism underlying the promotion of regeneration events by aOECs. The results indicated that aOECs promoted neurological function recovery by shifting microglia from M1 to M2. Of importance, the further data demonstrated that aOECs promoted LPS-induced microglial polarization from M1 to M2 through TREM2/nuclear factor kappa beta (NF-κB) signaling pathway. Finally, we found that apolipoprotein E (APOE) secretion was significantly elevated in aOECs, indicating that APOE secreted by aOECs might participate in regulating TREM2. Taken together, these findings demonstrated that aOECs could improve neurological function after SCI by switching microglial polarization through APOE/TREM2/NF-κB signaling pathway.

## Methods

### Animals and Experimental Protocol

All experimental protocols involved in this study were reviewed and approved by the Ethics Committee for the Experimental Animals of Hong Hui Hospital, Xi’an Jiaotong University. All animals used in this study were supplied by the Laboratory Animal Center of Xi’an Jiaotong University. In total, 60 Sprague Dawley (SD) female rats, weighing approximately 220 g, were randomly allocated to the following four experimental groups: (i) in the sham group, rats only suffered laminectomy only at the T8-10; (ii) in the control group, rats suffered from SCI surgery and were injected with the same volume saline; (iii) in the OECs group, rats suffered from SCI surgery and were transplanted with OECs; and (iv) in the aOECs group, rats suffered from SCI surgery and were transplanted with aOECs.

### Isolation, Culture and Identification of Primary OECs

Primary OECs were obtained from adult SD rat olfactory bulb (OB) at 2–3 months of age and were further purified by differential cell adhesiveness according to previous reports (Hao et al. 2017; Nash et al. 2001). In brief, the olfactory bulb was dissected and the superficial meninges and blood vessels were removed (Supplementary Fig. 1a). After removal of OB inner layer, the outer layer including olfactory nerve and glomerular layer was collected. Concomitantly, the tissues were rinsed thrice in HBSS, minced with iris scissor, and digested through 0.25% trypsin (Gibco, Carlsbad, CA, USA) at 37 °C for 25 min. When the trypsinization was terminated, the digested tissue was triturated and centrifuged at 1000 rpm/min for 5 min. Cells were resuspended in the Dulbecco’s Modified Eagle’s Medium (DMEM)/F12 (Gibco, Carlsbad, CA, USA) containing 20% fetal bovine serum (FBS; Gibco, Carlsbad, CA, USA) and 1% penicillin-streptomycin (Sigma-Aldrich, St. Louis, MO, USA), seeded into flasks coated with poly-lysine (PLL; Sigma-Aldrich, St. Louis, MO, USA) and cultured at 37 °C incubator at 5% CO_2_. Culture medium was refreshed every three-four days. As for cell purification, the procedure was conducted according to previous reports (Hao et al. 2017). Lastly, the purity of OECs were determined by immunofluorescence staining with anti-p75 antibody and DAPI.

### Activation and Collection of Conditional Medium of OECs

To conduct the following experiments, OECs were reseeded on the 6-well plates or coverslips, and divided into two groups including the activated and control groups. For activated group, OECs were treated with 1µM CCM (Sigma-Aldrich, St. Louis, MO, USA). For control group, OECs were treated with equal volume of DMSO vehicle (Sigma-Aldrich, St. Louis, MO, USA). As for collection of conditioned medium, in order to eliminate the nonspecific effects of CCM or DMSO itself, the culture supernatant of both groups was collected at 24, 48 and 72 h, respectively after culture medium was replaced with the fresh medium at 3 days post treatment with CCM. Lastly, the harvested conditional media were mixed, centrifuged to eliminate cell debris and stored at -80℃ for further experiments.

### Culture and Purification of Primary Microglia

Primary microglia were obtained from 3-day-old SD rat brain as described previously (Ji et al. 2019). Briefly, the cortices were dissected, removed the superficial meninges and blood vessels and thoroughly rinsed with ice-cold HBSS (Supplementary Fig. 1b, c). Concomitantly the tissues were minced and digested with 0.25% trypsin for 20 min. After the digestion was terminated, the digested tissues were triturated and centrifuged at 1000 rpm for 5 min. The dissociated cells were resuspended with DMEM (Gibco, Carlsbad, CA, USA) containing 10% FBS and 1% penicillin-streptomycin. The cells were seeded into PLL-coated flask and cultured in an incubator at 37 °C in a humidified atmosphere of 5% CO_2_. Half of medium was replaced every three days until cells reached over 85% confluence. The cell-grown flask was fixed on a shaker and continuously shaken for 5 h at 260 rpm to harvest microglia. The purity of microglia was identified by immunostaining with anti-Iba1 antibody.

### SCI Model and Cell Transplantation

The rat SCI models were developed according to previous publication (Fan et al. 2019). In brief, the adult SD rats weighing 220 g were firstly anesthetized by intraperitoneal injection of 1% sodium pentobarbital (60 mg/kg). After hair removal and disinfection, a laminectomy was performed at T8-10 to expose the underlying spinal cord the tissues. The spinal cord at T9 was then clamped with forceps (53327T, 66 Vision-Tech Co., Ltd.) for 20 s. Notably, the parallel distance of forceps tip was kept at 0.5 mm width when the spinal cord was crushed.

For cell transplantation, 2 µL cell suspension containing 1 × 10^5^ OECs or aOECs were immediately injected into the core site of the injured spinal cord through 10 µL siliconized Hamilton syringe post-injury. The syringe was fixed on the stereotaxic instrument to maintain a uniform rate of 0.2 µL/min. In control group, the equal volume saline was injected using the same method. Notably, in order to trace the growth and migration of OECs in vivo, OECs obtained from enhanced green fluorescent protein (EGFP) transgenic rats, treated with CCM, and transplanted into SCI models (n = 3/group), respectively. As for other experiments, OECs/aOECs were derived from normal SD rats.

After surgery, antibiotics, penicillin (1 × 10^4^ U) and gentamicin (8 × 10^4^ U), were administered subcutaneously to prevent infection (Guo et al. 2020). Meanwhile, manual-assisted urination was performed until the recovery of micturition function.

### Assessment of Behavior

Functional assessment was performed with the Basso, Beattie, and Bresnahan (BBB) locomotor scale reported by Basso et al. (1995). The BBB scores of each rat were recorded at preoperative 1 day and 1, 3, 7, 14, 21 and 28 days after transplantation. The scores were observed and recorded independently by two trainees blinded to the allocation of experimental groups.

### Treatment of Primary Microglia

Microglia were reseeded on the 6-well plates or coverslips, and divided into the following groups: control, LPS, LPS + OECs-CM, and LPS + aOECs-CM groups. Except for the control group, the other groups were treated with medium containing 100 ng/mL LPS (Sigma-Aldrich, St. Louis, MO, USA) for 24 h and the medium was replaced with normal medium, OECs-CM and aOECs-CM, respectively. Forty-eight hours later, cells were collected to perform immunofluorescence and extract total protein for western blot.

### Small Interfering RNA (siRNA) Transfection of Microglia

To examine the involvement of TREM2 in microglial polarization, microglia grown in the 6 well plates were transfected with siRNA (GenenPharma, Shanghai, China) targeting TREM2 using Lipofectamine® 3000 Transfection Reagent (Invitrogen, NY, USA) according to the instruction of manufacturer. The sequences are displayed in the Supplementary Table 1. Forty-eight hours later, the total proteins were extracted to assess the efficiency of siRNA knockdown by western blot analysis.

### Western Blot Analysis

At the termination of different treatments, cells or spinal cord tissues (2.0 mm above and below the lesion, Supplementary Fig. 2) were collected to extract total proteins for western blot as the previously described (Wang et al. 2022; Guo et al. 2020). In this study, the following specific antibodies were used: TG2 (3557, 1:1000, Cell Signaling Technology), PSR(PAB916Hu01, 1:1000, Cloud-Clone Corp.), iNOS (ab49999, 1:2000, Abcam), Arg-1 (ab60176, 1:3000, Abcam), CD86 (91882, 1:2000, Cell Signaling Technology), CD206 (60143-1-Ig, 1:2000, Proteintech), TREM2 (ab95470, 1:1500, Abcam), pNF-κB (3033, 1:2000, Cell Signaling Technology), APOE (ab183597, 1:2000, Abcam) and β-actin (T0022, 1:5000, Affinity). After primary antibodies overnight at 4 °C, the corresponding secondary antibodies were incubated based on the manufacturer’s instructions. The immunoblots were visualized using ECL kit and scanned by ChemiDoc XRS (Bio-Rad, CA, USA). Image J software was used to analyze the intensity of the bands based on the β-actin level.

### Immunocytofluorescence Staining

OECs and microglia grown on the coverslips were treated according to the experimental protocols. Then the immunofluorescence was performed following previously described methods (Wang et al. 2022; Guo et al. 2020). The primary antibodies were used to stain cells, including anti-p75 (ab245134, 1:300, Abcam), Iba1 (019-19741, 1:500, Wako), iNOS (ab49999, 1:300, Abcam), Arg-1 (ab60176, 1:300, Abcam), TREM2 (ab95470, 1:500, Abcam), F4/80(ab6640, 1:200, Abcam), pNF-κB (3033, 1:200, Cell Signaling Technology), APOE (ab183597, 1:200, Abcam) and DAPI (ab228549, 1:1000, Abcam). After incubation with the corresponding secondary antibodies, coverslips were washed and mounted on the slides. All images were captured by fluorescence microscope (Leica Microsystems, Germany).

### Immunohistofluorescence Staining

For immunohistofluorescence staining, rats were anesthetized and transcardially perfused with 200 mL 0.9% saline, followed by 400 mL 4% paraformaldehyde at seven days post-transplantation (Supplementary Fig. 3a). Thereafter, the spinal cord was dissected (1.0 cm above and below the injured site, Supplementary Fig. 3b) and cryoprotected in 30% sucrose in 0.1 M PBS dehydrate at 4 °C. The tissues were sliced at 10 mm thickness by cryostat and fixed on the PLL-coated slides. The details of immunohistofluorescence staining were consistent with the above-mentioned description (Fan et al. 2019). The primary antibodies included anti-p75 (ab245134, 1:200, Abcam), GFP (ab6673,1:200, Abcam), Iba1 (019-19741, 1:500, Wako), iNOS (ab49999, 1:200, Abcam), Arg-1 (ab60176, 1:200, Abcam), NF68 (2835s, 1:50, Cell Signaling Technology) and DAPI (ab228549, 1:1000, Abcam). Other following procedures were same as the immunocytofluorescence.

### Enzyme-linked Immuno Sorbent Assay (ELISA)

To explore the potential mechanism, ELISA was used to detect the APOE concentration of conditional medium from culture of OECs and aOECs. The procedures were performed in accordance with the guidelines of ELISA kit (Elabscience®, E-EL-R1230c, Wuhan, China). After reaction termination, the optical density (OD) was measured in a microplate reader at 450 nm wavelength. Standard curve was established according to OD value of standard samples and then calculated the concentration of APOE based on OD of samples.

### Statistical Analysis

All experimental data were obtained from at least three independent repetitions and presented as the mean ± standard deviation (SD). SPSS 23.0 software (IBM, NY, USA) was used to perform statistical analysis. Student’s *t*-test were used to compare the difference between the two groups. The significance of multiple groups was determined by analysis of variance. Statistical significance was defined as *P* value<0.05. Graph Prism 8.0 (GraphPad Software, CA, USA) was used to prepare graphs based on the results of statistical analysis.

## Results

### Morphological Characteristics and Identification of OECs and Microglia

**Fig. 1:**
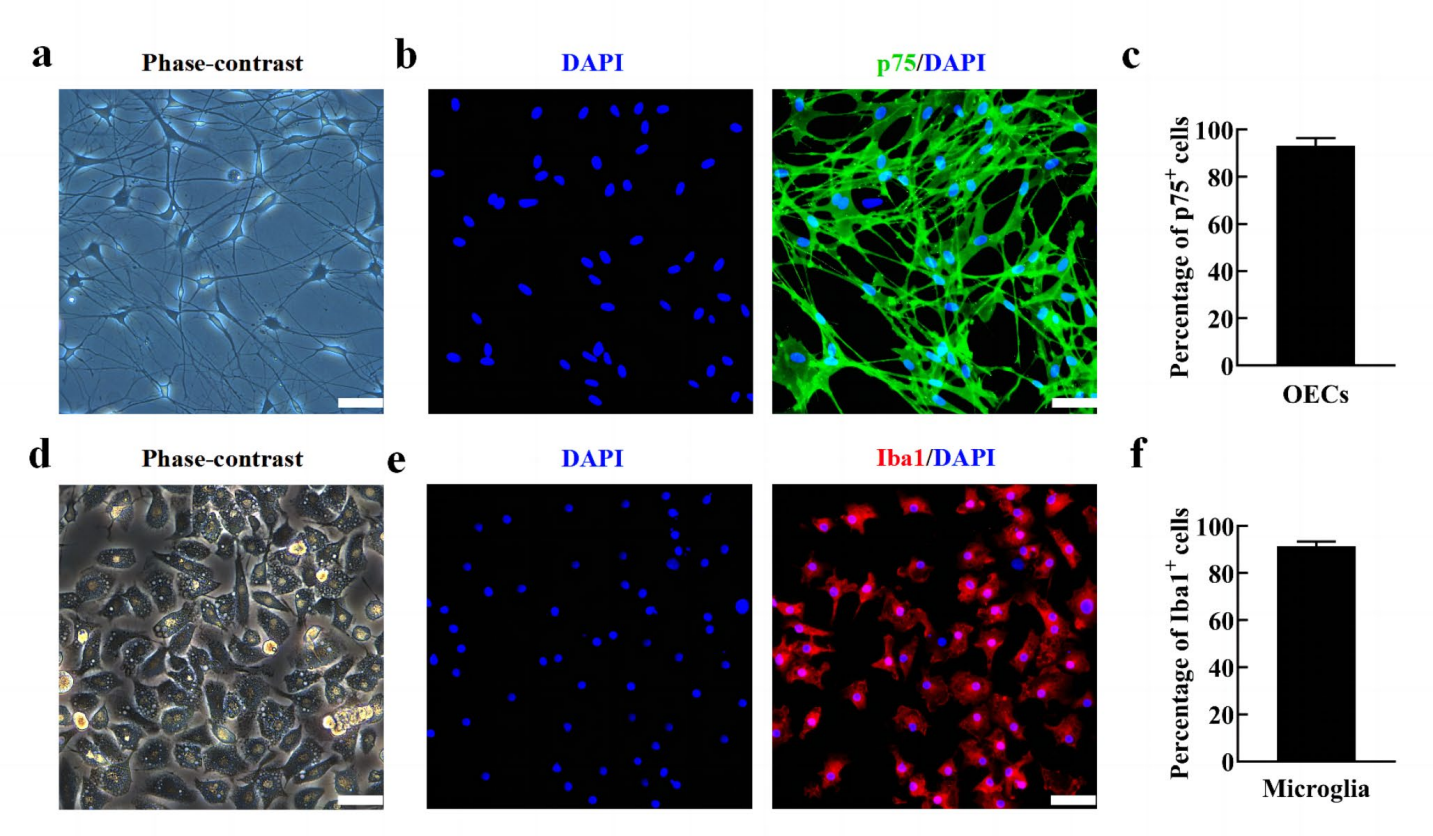
Morphological characteristics and identification of OECs and microglia. **(a)** Typical morphological characteristics of primary OECs under the phase-contrast microscope at 9 days. **(b)** Immunocytofluorescence staining for p75 (Scale bar = 50 μm). **(c)** Quantification of p75 positive cells in the primary OECs (Note: the p75^+^ cells were regarded as OECs, the purity of OECs = p75^+^/DAPI^+^%, n = 6). **(d)** Representative morphological features of primary mciroglia under the phase-contrast microscope. **(e)** Immunocytofluorescence staining for Iba1 (Scale bar = 100 μm). **(f)** Quantification of Iba1 positive cells in primary microglia (Note: the Iba1^+^ cells were regarded as microglia, the purity of microglia = Iba1^+^/DAPI^+^%, n = 6)

To investigate whether aOECs potentiates the polarization of microglial subtypes, OECs were first cultured and identified using the above-described in method part. The typical morphological characteristics of primary OECs after purification at 7–10 days was evaluated by phase-contrast microscope. As shown in Fig. 1a, the majority of OECs bodies showed flat and unipolar, bipolar or multipolar state with long and slender neurites. The purified OECs was almost positive for p75 and the proportion of p75 positive cells accounted for approximately 93% (Fig. 1b, c). Notably, OECs proliferation was evaluated after CCM treatment at different time points. The results showed that CCM significantly promoted OECs proliferation at 3 days after treatment compared with control group (Supplementary Fig. 4a, n = 3, *P<0.05, **P<0.01, compared with control group), indicating that CCM enhanced OECs activation. To further verify if CCM could effectively elicit the activation of OECs, the representative markers involved cell activation, TG2 and PSR, were detected using western blot. The results showed that the expression of TG2 and PSR were significantly increased in OECs after 1, 2 and 3 days of CCM treatment (n = 3, *P<0.05, **P<0.01, ***P<0.001, compared with control group, Supplementary Fig. 4b-d), suggesting that CCM can evoke OECs activation.

Microglia cells reached optimal confluence after being purified and displayed a small and irregular shape with short spines and cytoplasm contained substantial particles (Fig. 1d), which were similar to macrophage morphological features. Moreover, the immunocytofluorescence staining indicated that the percentage of positive cells for Iba1 were more than 90% (Fig. 1e, f), suggesting that we obtained high purity of microglia.

### Transplantation of aOECs Improved the Neurological Function Post SCI

**Fig. 2:**
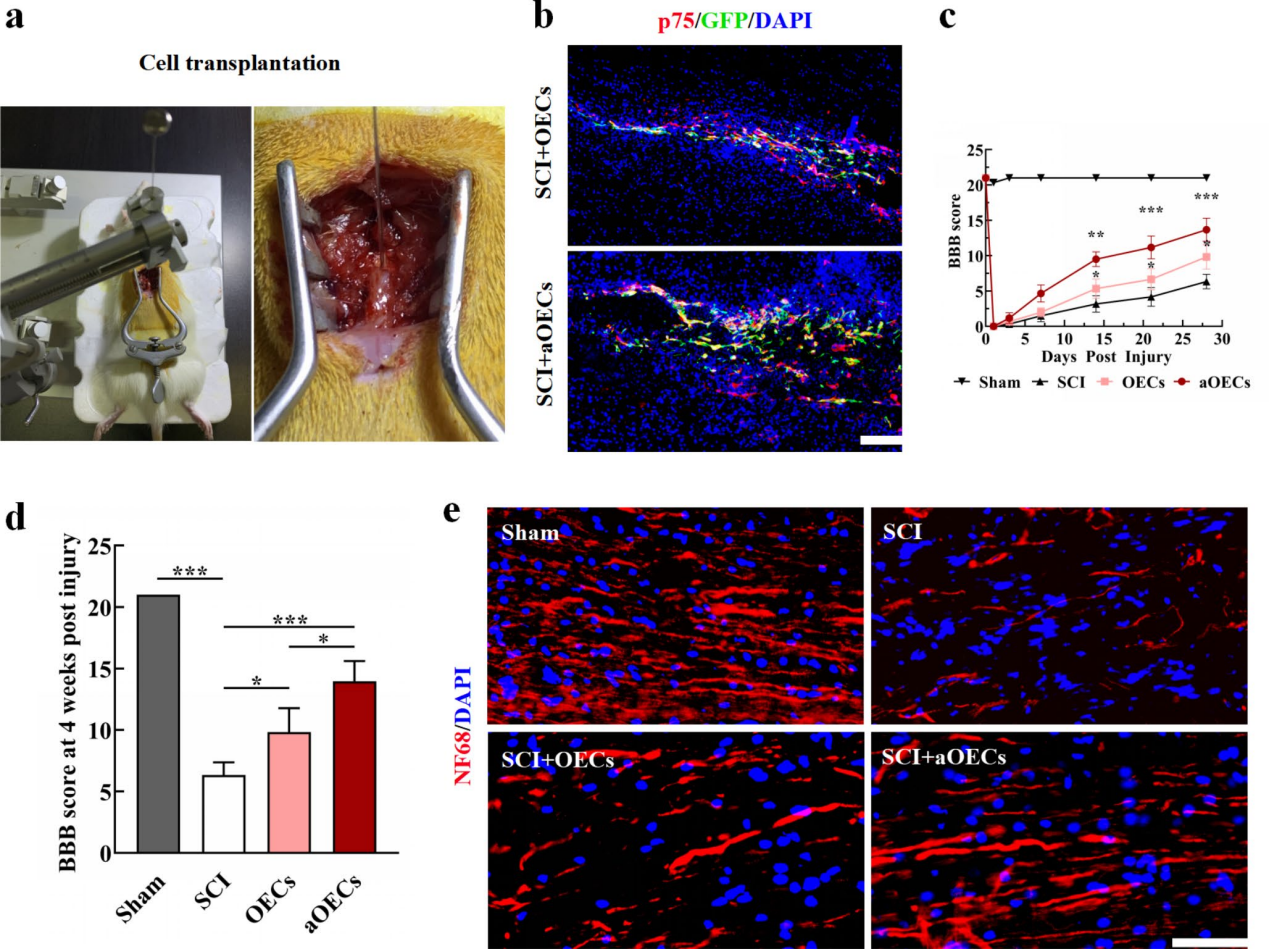
Transplantation of aOECs improved the neurological function post SCI. **(a)** Injection of OECs into the injured site after SCI. **(b)** Immunohistofluorescence staining for p75 and GFP at 1 week post-injury (Scale bar = 50 μm). **(c)** BBB scores at different time points in each group (n = 6/group, *P<0.05, **P<0.01, ***P<0.001, compared with SCI group). **(d)** BBB scores at 4 weeks post injury in each group (n = 6/group, *P<0.05, ***P<0.001, compared with SCI group; *P<0.05, SCI + aOECs compared with SCI + OECs group). (e) The distribution of nerve fibers in the indicative group (Scale bar = 100 μm)

To evaluate whether transplantation of aOECs exerted beneficial effects on SCI rats, we established SCI models and transplanted aOECs into the injured spinal cord (Fig. 2a). According to the time point of experimental designs, we further carried out immunohistofluorescence staining to observe migration and survival of OECs in the lesioned spinal cord tissues. As shown in Fig. 2b, the majority of cells were co-labeled with p75 and GFP and migrated to the regions distant from transplant sites, indicating that the implanted OECs were high purity and viability. Behavioral measurements after cells transplantation were made by BBB scores at 1, 3, 7, 14, 21 and 28 days post-surgery. We found that, besides from sham group with transient locomotor disorder on the day of injury, other groups showed obviously locomotor disability and gradually recovered over time, which was similar to previous studies (Wang et al. 2022; Zhang et al. 2022). Compared with SCI group, both aOECs-transplanted group and OECs-transplanted group showed significantly higher BBB scores, displaying excellent effects (n = 6, *P<0.05, **P<0.01, ***P<0.001, Fig. 2c, d). Intriguingly, the BBB scores of aOECs-transplanted group were greater than OECs-transplanted group at 4 weeks (n = 6, *P<0.05, Fig. 2d), indicating that aOECs may be more potential for treatment SCI. In addition, immunohistofluorescence staining for NF68 showed that NF68-immunopositive nerve fibers were well distributed in sham group at 4 weeks, but were broken, sparse and irregular in SCI group. For aOECs-transplanted and OECs-transplanted groups, nerve fibers exhibited relative continuity and rostral-caudal directionality to some extent, yet the former was superior to the latter regarding the number of intact nerve fibers (Fig. 2e). These findings suggested that transplantation of aOECs ameliorated neurological function in SCI rats.

### The Effect of aOECs on Microglia Polarization After SCI

**Fig. 3:**
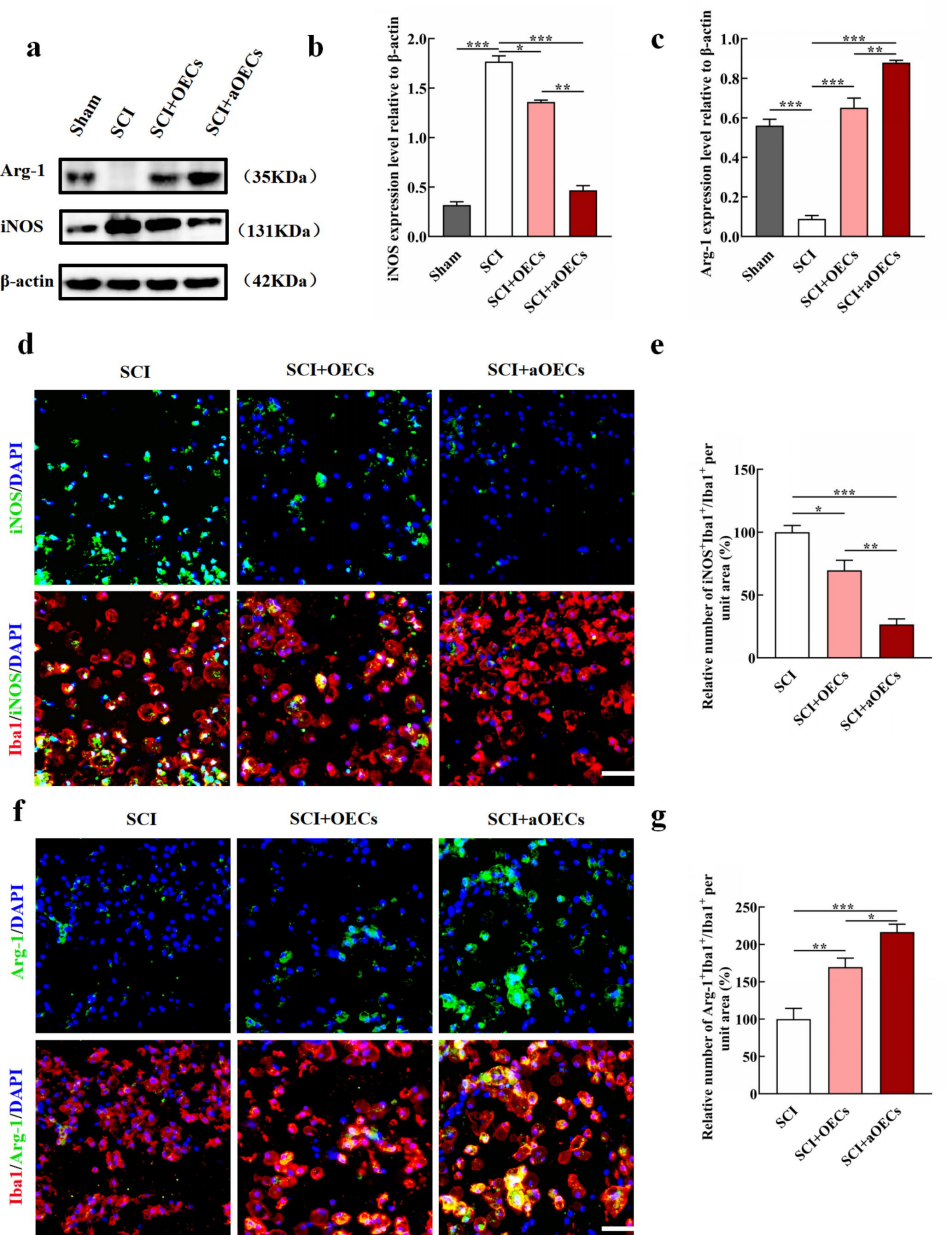
The effects of aOECs on microglia polarization after SCI. **a.** Western blot analysis of iNOS and Arg-1 expression in the lesioned spinal cord in sham, SCI, SCI + OECs and SCI + aOECs group at 1 week after SCI. **b-c.** Quantification of iNOS and Arg-1 relative exprseeion in each group (n = 3, *P<0.05, ***P<0.001, compared with SCI group; **P<0.01, SCI + aOECs compared with SCI + OECs group). **d.** Immunohistofluorescence of iNOS and Iba1 in SCI, SCI + OECs and SCI + aOECs group at 1 week after SCI (Scale bar = 100 μm). **e.** Quantification of iNOS-positive microglia in the lesion site (n = 3, *P<0.05, ***P<0.001, compared with SCI group; **P<0.01, SCI + aOECs compared with SCI + OECs group). **f-g.** Immunohistofluorescence of Arg-1/Iba1 (Scale bar = 100 μm) and quantification of Arg-1-positive microglia in indicated treatments at 1 week after SCI (n = 3, **P<0.01, ***P<0.001, compared with SCI group; *P<0.05, SCI + aOECs compared with SCI + OECs group).

Given that inflammatory microenvironment plays crucial roles in functional recovery after SCI and aOECs show excellent anti-inflammatory properties (Guo et al. 2020; Fan et al. 2022; Jiang et al. 2022a, b), we further evaluated the effects of aOECs on inflammation mediated by microglia. Providing a permissive environment after SCI by switching microglia polarization from M1 to M2 is extremely favorable (Kobashi et al. 2020; Liu et al. 2020). Accordingly, we speculated whether aOECs could inhibit inflammation through regulating microglia polarization. To confirm the roles of aOECs in modulating microglial phenotype, we investigated the expression level of iNOS and Arg-1 at one week after transplantation aOECs. The results indicated that iNOS expression in SCI group was significantly higher than that in sham group, OECs and aOECs groups. Also, quantification analysis revealed that there was a significant difference among these groups (n = 3, *P<0.05, **P<0.01, ***P<0.001, Fig. 3a, b). Intriguingly, aOECs group showed significantly pronounced inhibition in iNOS expression compared with OECs group. Reversely, Arg-1 expression in injured area was highest in aOECs group (n = 3, **P<0.01, ***P<0.001, Fig. 3a, c). To further validate our findings, immunohistofluorescence double staining for Iba1 and iNOS or Arg-1 was performed in the lesioned spinal tissues. Similar to the results of western blot, the immunohistofluorescence indicated that iNOS-positive microglia remarkably decreased in the injured area compared with SCI group (n = 3, *P<0.05, **P<0.01, ***P<0.001, Fig. 3d, e), whereas the Arg-1-positive mciroglia significantly increased (n = 3, *P<0.05, **P<0.01, ***P<0.001, Fig. 3f, g). Taken together, these results suggested that aOECs suppressed the inflammatory responses via polarizing microglia to M2 phenotype.

### aOECs Promoted Microglia Polarization to M2 Phenotype in Vitro

**Fig. 4:**
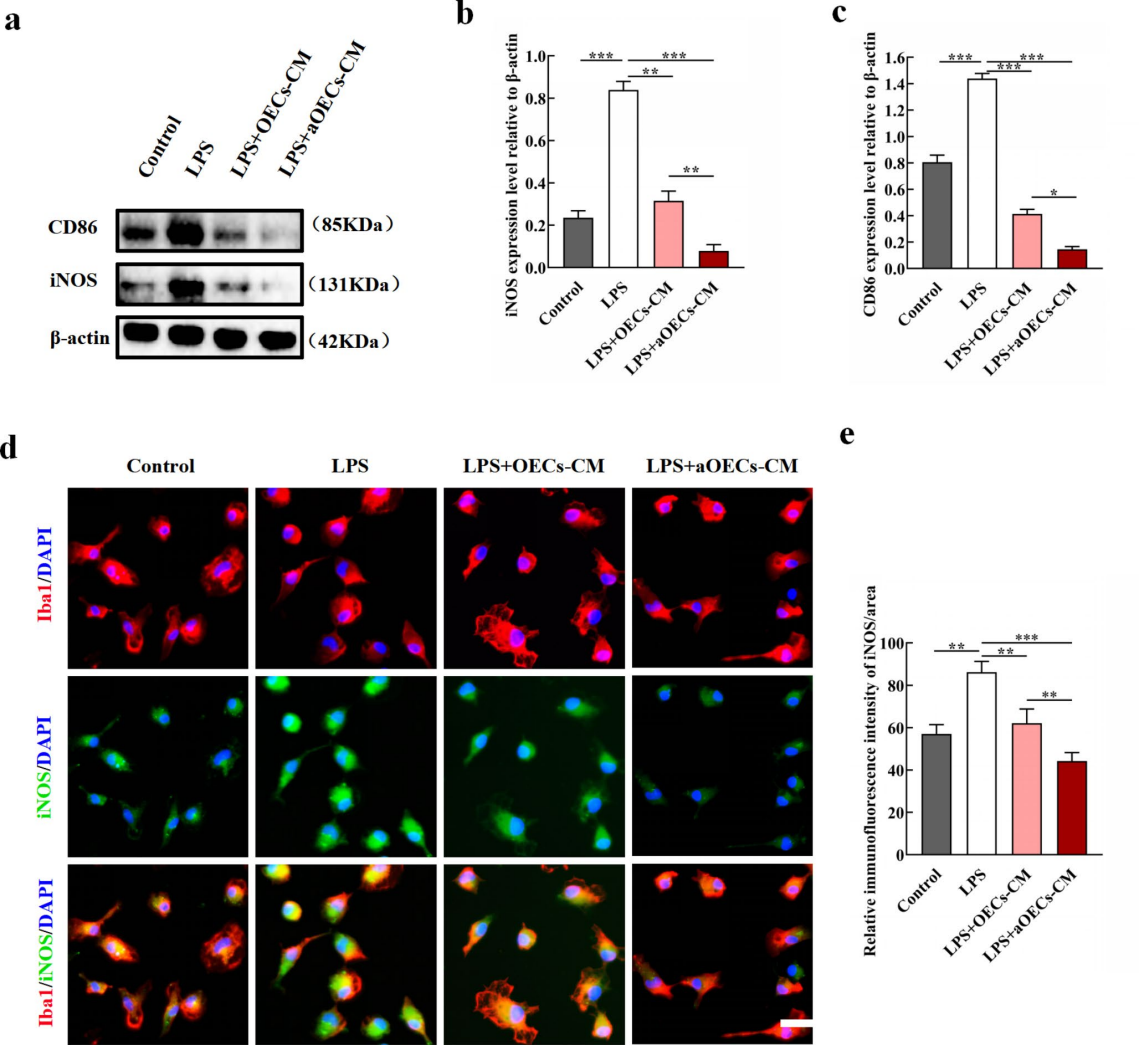
The inhibition of aOECs on LPS-induced microglia polarization to M1. **a.** Western blot analysis of iNOS and CD86 expression level in microglia with indicated treatments. **b-c.** Quantification of iNOS and CD86 relative expression in microglia with indicated treatments (n = 3, **P<0.01, ***P<0.001, compared with LPS group; *P<0.05, **P<0.01, LPS + aOECs-CM compared with LPS + OECs-CM group). **d.** Immunocytofluorescence staining of iNOS and Iba1, respectively (Scale bar = 50 μm). **e.** Quantification of iNOS immunofluorescence intensity in each group (n = 3, **P<0.01, ***P<0.001, compared with LPS group; **P<0.01, LPS + aOECs-CM compared with LPS + OECs-CM group)

To further substantiate the claim regarding aOECs promoting microglia polarization, microglia polarization to M1 was induced and then treated by OECs-CM and aOECs-CM in vitro. The data indicated that aOECs-CM decreased the pro-inflammatory factors level of iNOS and CD86 expressed in LPS-mediated M1 phenotypic microglia (n = 3, *P<0.05, **P<0.01, ***P<0.001, Fig. 4a-c). In addition, immunocytofluorescence staining showed that aOECs-CM significantly suppressed the elevated expression of iNOS caused by LPS in primary microglia (n = 3, **P<0.01, ***P<0.001, Fig. 4d, e). These data implied that aOECs-CM inhibited LPS-induced microglia polarization to M1.

**Fig. 5:**
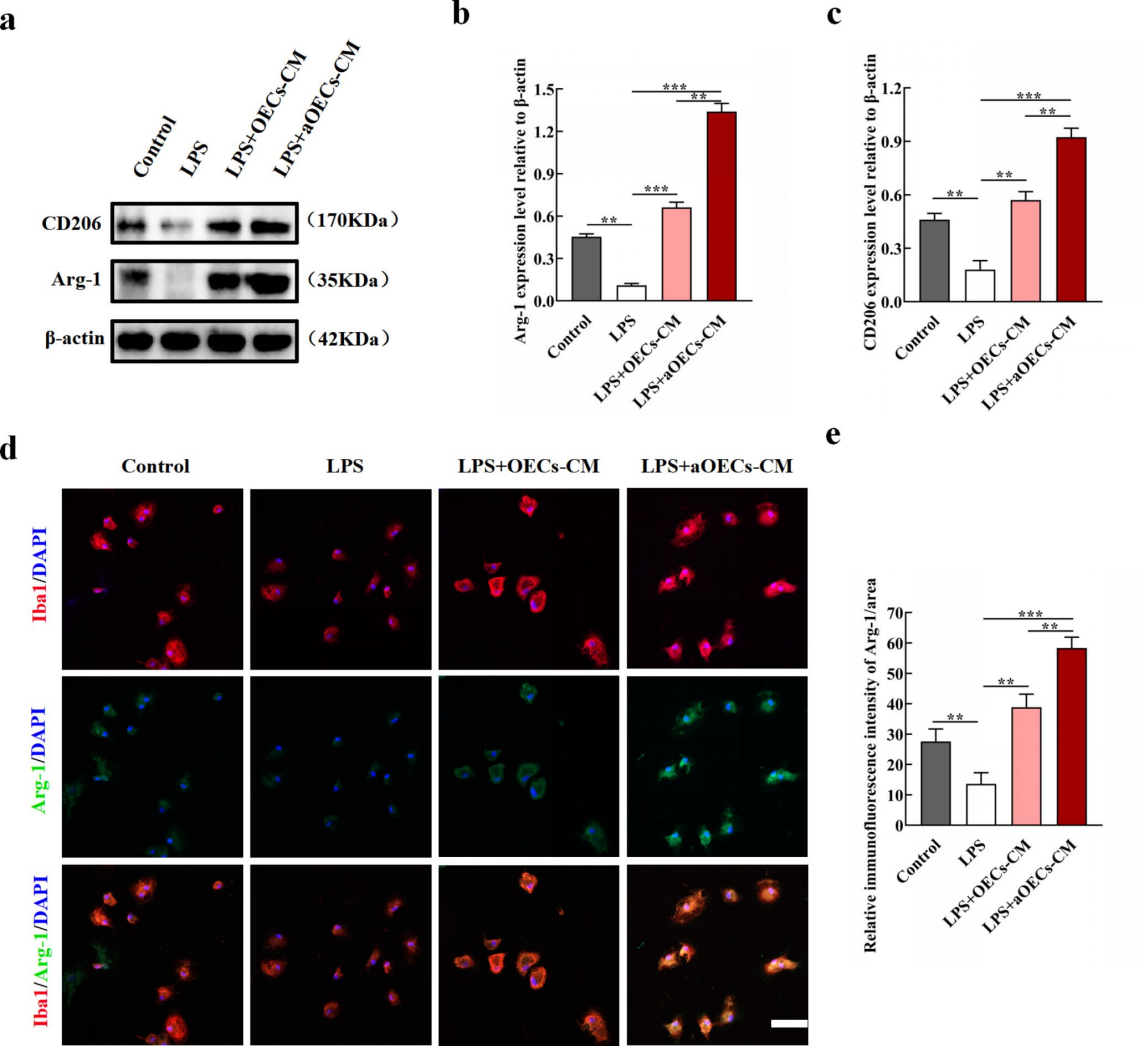
aOECs promoted microglia polarization to M2 in LPS-induced microglia. **a.** Western blot analysis of Arg-1 and CD206 expression level in microglia in indicted treatments. **b-c.** Quantification of Arg-1 and CD206 relative expression in microglia in indicted treatments (n = 3, **P<0.01, ***P<0.001, compared with LPS group; **P<0.01, LPS + aOECs-CM compared with LPS + OECs-CM group). **d.** Immunocytofluorescence staining of Arg-1 and Iba1, respectively (Scale bar = 100 μm). **e.** Quantification of Arg-1 immunofluorescence intensity in each group (n = 3, **P<0.01, ***P<0.001, compared with LPS group; **P<0.01, LPS + aOECs-CM compared with LPS + OECs-CM group)

Furthermore, the anti-inflammatory markers were examined to determine the effects of aOECs on M2 polarization. With regard to the anti-inflammatory makers of Arg-1 and CD206, the expression significantly increased after treatment with aOECs-CM in LPS-induced primary microglia (n = 3, **P<0.01, ***P<0.001, Fig. 5a-c). Simultaneously, immunocytofluorescence showed that aOECs significantly reversed the decrease of immunofluorescence intensity of Arg-1 in primary LPS-induced microglia (n = 3, **P<0.01, ***P<0.001, Fig. 5d, e). Therefore, aOECs effectively inhibited LPS-induced microglial polarization to M1 and facilitated microglia phenotype toward M2 polarization.

### TREM2/NF-κB Participated in the Phenotypic Change of Primary Microglia

**Fig. 6:**
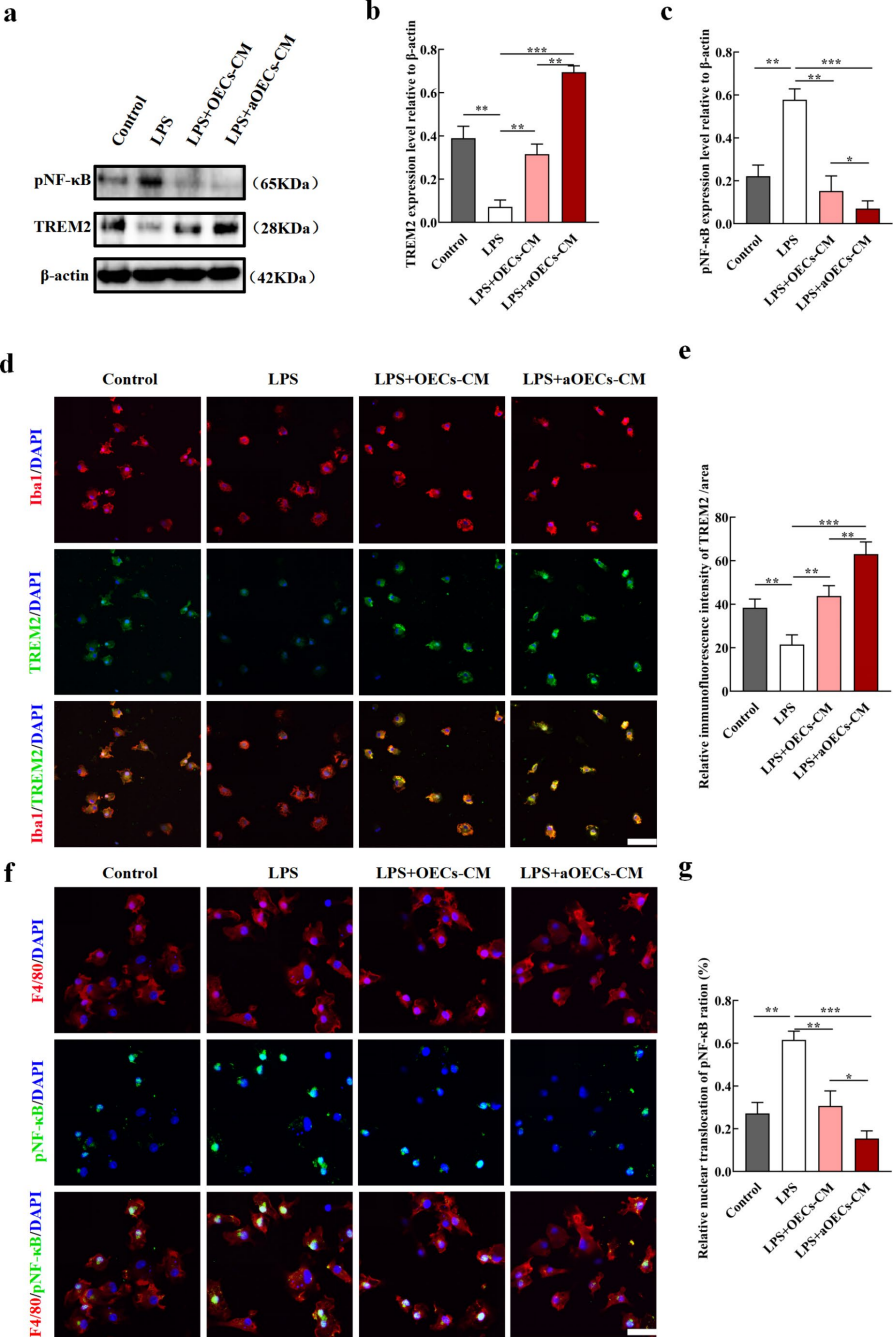
The possible mechanism underlying aOECs modulating microglia polarization from M1 phenotype to M2. **a.** Western analysis of TREM2 and pNF-κB expression level in LPS-induced microglia treated by OECs-CM and aOECs-CM. **b-c.** Quantification of TREM2 and pNF-κB relative expression in microglia under indicated treatments (n = 3, **P<0.01, ***P<0.001, compared with LPS group; *P<0.05, **P<0.01, LPS + aOECs-CM compared with LPS + OECs-CM group). **d.** Immunocytofluorescence staining of TREM2 and Iba1 in each group (Scale bar = 75 μm). **e.** Quantification of TREM2 immunofluorescence intensity (n = 3, **P<0.01, ***P<0.001, compared with LPS group; **P<0.01, LPS + aOECs-CM compared with LPS + OECs-CM group). **f.** Immunocytofluorescence staining of pNF-κB and F4/80 in microglia with indicated treatments (Scale bar = 50 μm). **g.** Quantification of the relative nuclear translocation of pNF-κB ration (n = 3, **P<0.01, ***P<0.001, compared with LPS group; *P<0.05 LPS + aOECs-CM compared with LPS + OECs-CM group)

Compelling evidences demonstrated that TREM2 plays crucial roles in immunemodulating of microglia and is closely related to microglia polarization (Zhai et al. 2017; Sanjay, Shin et al. 2022; Piccio et al. 2007). Whether TREM2 is involved in the potential mechanism of aOECs-mediated microglia polarization is still unknown. As we speculated, western blot revealed that TREM2 expression of remarkably was increased in LPS-induced microglia following the administration of aOECs-CM (n = 3, **P<0.01, ***P<0.001, Fig. 6a, b). Consistently, TREM2 immunofluorescence intensity in microglia treated with aOECs-CM significantly was enhanced (n = 3, **P<0.01, ***P<0.001, Fig. 6d, e). Considering that NF-κB signaling pathway exerted substantial effects on inflammatory cascades, we further analyzed the effects of aOECs on NF-κB, and found that LPS-induced NF-κB activation was significantly inhibited by aOECs-CM (n = 3, *P<0.05, **P<0.01, ***P<0.001, Fig. 6a, c). For better understanding the NF-κB activation, we investigated the nuclear translocation of pNF-κB by immunofluorescence staining for F4/80 and pNF-κB. The results showed that aOECs-CM remarkably suppressed the nuclear translocation of pNF-κB in the LPS-induced microglia (n = 3, **P<0.01, ***P<0.001, compared with LPS group; *P<0.05, LPS + aOECs-CM compared with LPS + OECs-CM group, Fig. 6f,g). These data indicated that aOECs modulated microglial polarization partially through the TREM2/NF-κB axis.

### Knockdown of TREM2 Reversed aOECs-mediated Microglia Polarization to M2

**Fig. 7:**
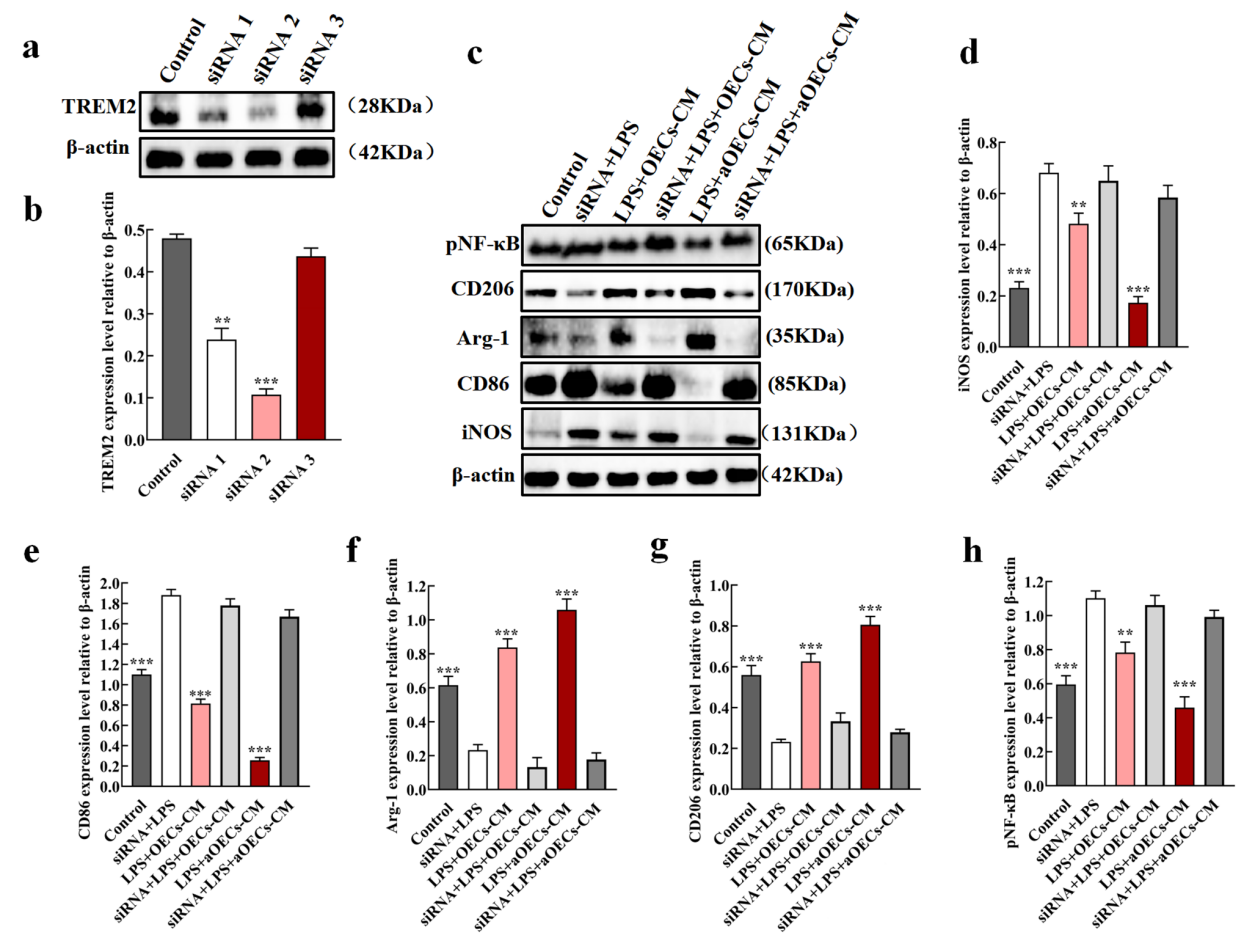
TREM2 Knockdown reversed aOECs-CM mediated microglia polarization to M2. **a.**Western blot analysis of TREM2 expression after transfection siRNA. **b.** Quantification of TREM2 relative expression (n = 3, **P<0.01, ***P<0.001, compared with control group). **c.** The expression of M1 phenotypic markers (iNOS, CD86), M2 phenotypic markers (Arg-1, CD206) and pNF-κB after TREM2 knockdown among each group. **d-h.** Quantification of iNOS, CD86, Arg-1, CD206 and p65NF-κB relative expression (n = 3, **P<0.01, ***P<0.001, compared with siRNA+ LPS group)

To further substantiate the key role of TREM2 in switching microglia polarization, TREM2 knockdown was conducted to silence TREM2 mRNA and protein expression by siRNA specifically targeting TREM2 and evaluated its effects. The results obtained from western blot showed that TREM2-siRNA significantly reduced the expression of TREM2 (n = 3, **P<0.01, ***P<0.001, Fig. 7a, b). Consequently, the optimal efficiency of siRNA was selected to perform the subsequent experiments. Next, we assessed the expression of M1 and M2 markers following TREM2 knockdown. As shown in Fig. 7c, western blot showed that TREM2 knockdown resulted in up-regulation of M1 markers iNOS and CD86, and down-regulation of M2 markers Arg-1 and CD206, indicating that aOECs-mediated microglia polarization towards M2 in LPS-induced microglia was effectively reversed. Consistently, quantitative analysis revealed that there were statistically significant differences among each group (n = 3, **P<0.01, ***P<0.001, compared with siRNA + LPS group, Fig. 7d-g), further verifying that TREM2 deficiency resulted in failure of the positive role of aOECs in modulating microglia polarization toward M2 subphenotype. In addition, we explored the expression of NF-κB at the protein level in corresponding treatments after knockdown of TREM2 in vitro. The result showed that when TREM2 was knockdown in microglia, the expression of pNF-κB increased remarkably and there were significant differences among each group (n = 3, **P<0.01, ***P<0.001, compared with siRNA + LPS group, Fig. 7c, h). These results further verified that aOECs-CM polarized microglia towards M2 subtype by TREM2/NF-κB pathway.

### aOECs Promoted APOE Secretion

**Fig. 8:**
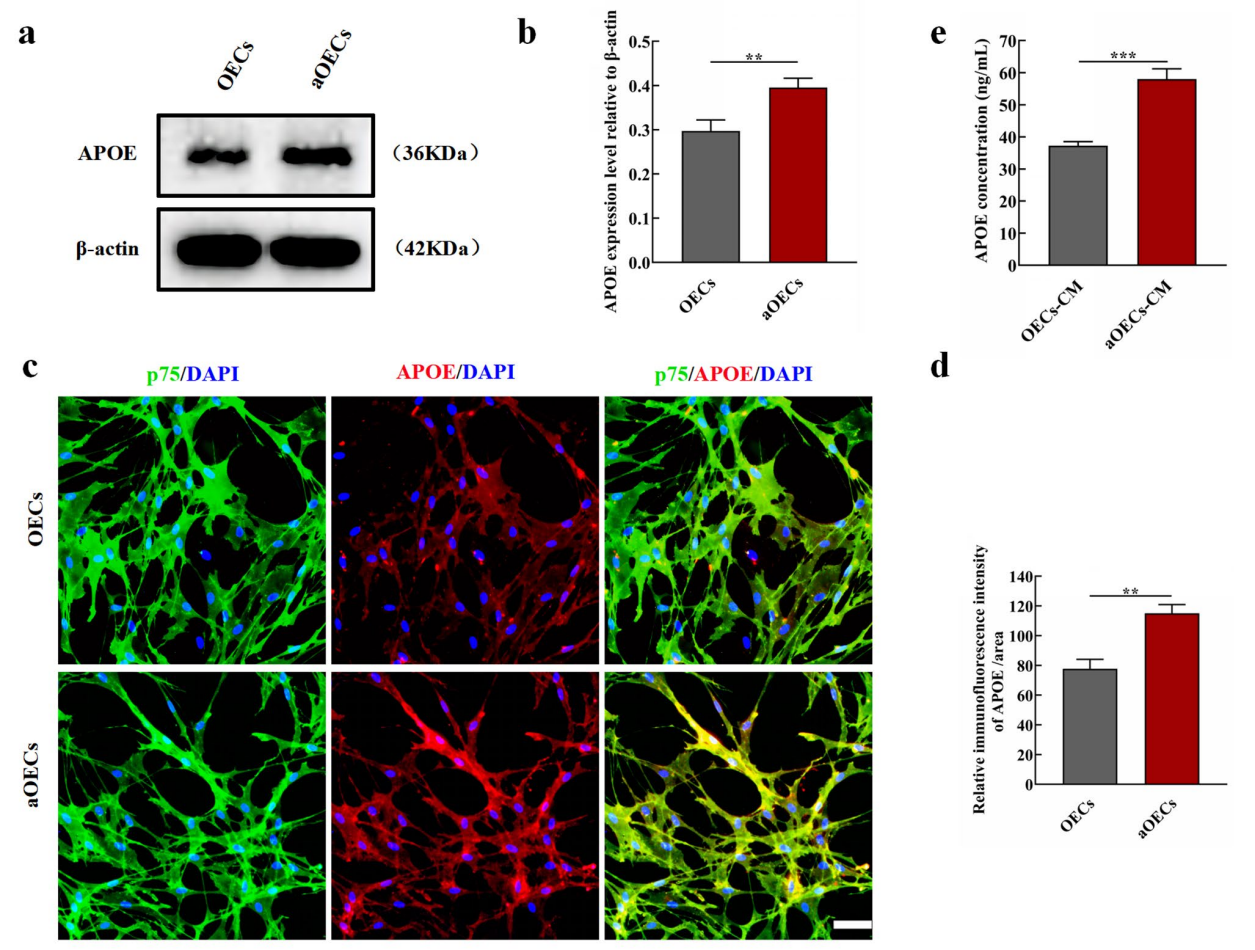
The effect of CCM on APOE secretion of OECs. **(a)** Western blot analysis of APOE expression level in OECs after different treatments. **(b)** Quantification of APOE relative expression in OECs treated with or without CCM (n = 3, **P<0.01, compared with OECs group). **(c)** Immunocytofluorescence staining for p75 and APOE in indicated groups (Scale bar = 50 μm). **(d)** Quantification of APOE immunofluorescence intensity (n = 3, **P<0.01, compared with OECs group). **(e)** The level of APOE in the supernatant of cultured OECs after treatment with CCM (n = 4, ***P<0.001, compared with OECs-CM group)

Although the present results reveal that aOECs modulate microglia polarization from M1 to M2 through TREM2/NF-κB signaling, the specific molecular mechanism by which aOECs trigger microglia polarization has not been fully elucidated. Previous studies reported that APOE acted as a ligand of multiple immune receptors and exerted immuneregulation (Mahoney-Sanchez et al. 2016; Wolfe et al. 2018). In addition, emerging evidence demonstrates that APOE, a high-affinity ligand for TREM2, could be recognized by TREM2, affecting microglia activation (Atagi et al. 2015; Yeh et al. 2016; Fitz et al. 2021). Thus, we analyzed the expression level of APOE in aOECs. Intriguingly, the expression of APOE significantly increased in aOECs treated with CCM when compared to that in OECs, (n = 3, **P<0.01, Fig. 8a, b). In addition, the immunocytofluorescence staining further indicated that CCM treatment triggered a higher expression of APOE in OECs (n = 3, **P<0.01, Fig. 8c, d). Considering that aOECs-CM was used to treat LPS-induced microglia in vitro, it is unclear whether aOECs-CM contains a higher concentration of APOE than OECs-CM. Therefore, the level of APOE was determined using ELISA assay. Our quantitative analysis showed that APOE concentration in aOECs-CM was significantly higher than that in OECs-CM (n = 4, ***P<0.001, Fig. 8e), suggesting that CCM significantly elevated APOE secretion of OECs.

## Discussion

SCI represents an extremely challenging issue in the clinical neurotrauma field mainly due to its multifaceted and dynamic pathological process (Yang et al. 2020; Afshari et al. 2009; Oyinbo 2011; Jiang et al. 2022a, b). To date, effective and feasible therapy strategies to overcome this overwhelming issue are still lacking. In this study, we found that aOECs could attenuate inflammatory response by regulating microglia from pro-inflammatory to anti-inflammatory phenotype in SCI rats, thus facilitating functional recovery. In addition, we demonstrated that aOECs played a favourable role in modulating LPS-induced microglia polarization to M2 through TREM2/NF-κB. Critically, CCM significantly elevated APOE production from OECs, which actively contributes to the cell events.

The detrimental microenvironment caused by massive inflammation has enormously negative impacts on neurological recovery after SCI (Anwar et al. 2016; David and Kroner 2011; Zhou et al. 2014). To the best of our knowledge, the pathological process of SCI involves both primary and secondary injuries, leading to a series of detrimental consequences (Anwar et al. 2016; Orr and Gensel 2018). In fact, secondary injury is the main contributor to the overall extent of SCI due to long-term existence in comparison with primary injury (Zhou et al. 2014). Inflammation, as the principal culprit, plays crucial role in the secondary injury, initiating the progressive aggravation of SCI. In addition, the inflammatory cascade triggered by immune cells produces large amounts of the cytokines and recruits inflammatory cells following SCI, which further exacerbates the microenvironment of the injured region (Orr and Gensel 2018; Xu et al. 2021). Microglia, as the resident immune cells in the CNS, are rapidly activated through damage-associated molecular patterns, leading to inflammation after SCI (Xu et al. 2021). Activated microglia is a double-edged sword with dual properties including beneficial and detrimental roles, which mainly relies on the pro-inflammatory or anti-inflammatory phenotypes (David and Kroner 2011; Xu et al. 2021). However, the pro-inflammatory phenotype (M1) usually predominate over anti-inflammatory phenotype (M2) after SCI, leading to a hostile microenvironment that is not conducive to neural regeneration (Kigerl et al. 2009). Consequently, it is essential to develop an effective approach to overcome the non-permissive environment through modulating the microglia phenotypes from M1 toward M2.

Currently, transplantation of OECs is regarded as a promising therapeutic strategy for SCI (Yang et al. 2015; Jiang et al. 2022a, b). OECs-based transplantation in SCI models has yielded great achievements. OECs implantation achieves neuroplasticity/neuroregeneration and improves neurological function by facilitating angiogenesis, axon outgrowth and remyelination in SCI (Gómez et al. 2018; Yang et al. 2015; Roet and Verhaagen 2014). More intriguingly, OECs possess unique characteristics such as immuno-modulation and debris-clearing activity conducive for neural regeneration (Zhang et al. 2019, 2021; Khankan et al. 2016; Saglam et al. 2021). In addition, OECs can be activated by CCM/LPS, heightening their bio-functions, including proliferation, migration and phagocytosis (Hao et al. 2017; Tello Velasquez et al. 2014). In addition, our latest study has indicated that activated OECs promote angiogenesis, and release neurotrophins and anti-inflammatory factors, resulting in superior neural regeneration outcomes (Guo et al. 2020). Nonetheless, the potential mechanism of aOECs transplantation resulting in neural regeneration after SCI through suppression of inflammation remains largely unknown. In the present study, aOECs transplantation exerted favorable effects on microglia polarization from M1 to M2, which may contribute to the mechanism of aOECs transplantation in SCI treatment.

To validate the effects of aOECs on microglia polarization, we induced microglia polarization to M1 with LPS and observed the effects of aOECs-CM in vitro. The results showed that aOECs-CM suppressed the expression of M1 markers in LPS-induced microglia and elevated the markers of M2, which were consistent with in vivo findings. However, the precise molecular mechanism underlying aOECs-mediated microglia polarization remains unclear. TREM2 is highly expressed in the cells of myeloid lineage, particularly in microglia, and plays extraordinary roles in immune regulation (Ford and McVicar 2009; Chen et al. 2020). Likewise, previous evidence revealed that TREM2 exerted robustly neuroprotective roles by anti-inflammation in several types of pathophysiological events, such as ischemic stroke, intracerebral hemorrhage and neurodegenerative diseases (Zhai et al. 2017; Chen et al. 2020; Jay et al. 2017). In addition, TREM2 deficiency exacerbates inflammation, while up-regulation of TREM2 exerts a positive role in alleviating neuroinflammation (Zhai et al. 2017; Piccio et al. 2007; Chen et al. 2020). Therefore, we also identified whether TREM2 is involved in aOECs-mediated microglia polarization events. Inspiringly, the further results showed that TREM2 expression significantly increased in LPS-induced microglia treated by aOECs-CM and NF-κB activation was remarkably suppressed, indicating that TREM2/NF-κB probably participated in aOECs-CM-modulated microglia polarization. For further substantiating the role of TREM2 in switching microglia polarization, we down-regulated TREM2 expression through siRNA. The data demonstrated that the expression of anti-inflammatory markers significantly decreased and pro-inflammatory factors increased after knockdown of TREM2, indicating that microglia polarization towards M2 caused by aOECs-CM in LPS-induced microglia was effectively reversed. Based on the above results, aOECs might switch LPS-induced microglia from M1 to M2 polarization through TREM2/NF-κB pathway.

APOE, a novel ligand with high-affinity for TREM2, exerts an important role in modulating microglial bio-function through APOE-TREM2 interaction (Atagi et al. 2015; Bailey et al. 2015; Yao et al. 2022). Therefore, we postulated that APOE released from aOECs was involved in regulating TREM2 expression, resulting in microglia phenotypic polarization. We noticed that APOE was expressed in OECs and the level of APOE in aOECs was higher than that in OECs. More significantly, aOECs-CM contained a higher amount of APOE than did OECs-CM, suggesting that CCM might strengthen APOE secretion from OECs. This result is in accordance with our recent finding that OECs activated by CCM strengthen their biofunctions. Specifically, recent study has demonstrated that the COG1410, an APOE-mimic peptide, strongly alleviates the neuroinflammation and neuronal apoptosis in mice with intracerebral hemorrhage, and TREM2/PI3K/Akt signal pathway involved in the cellular events (Chen et al. 2020). Moreover, it is reported that OECs can express APOE by means of immunostaining (Struble et al. 1999; Nathan et al. 2007). In combination with our in vitro data and these reports, we surmised that APOE released from OECs may bind TREM2 in microglia, leading to the activation of cascades responsible for microglia polarization. In addition, OECs can be effectively strengthened by CCM, causing the elevation of APOE secretion apart from several anti-inflammatory cytokines. On the basis of our present data as well as several previous reports, we speculate that boosting OECs biofunctions conducive to neural regeneration and functional recovery may, in part, be ascribed to promoting microglia polarization mediated by APOE/TREM2/NF-κB signaling pathway.

**Fig. 9:**
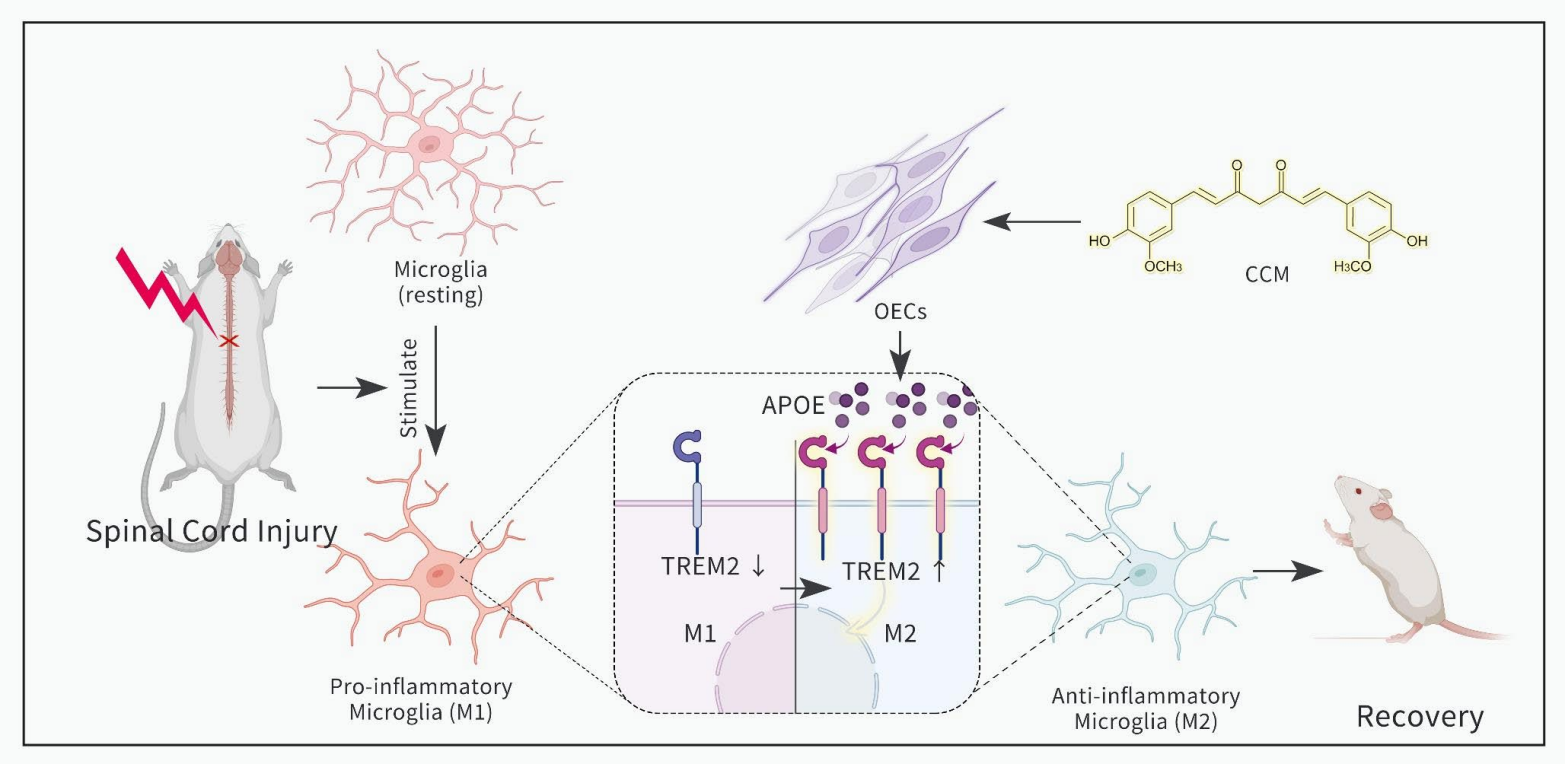
Schematic diagram displaying the outline of our findings. CCM-activated OECs could alleviate inflammation after SCI by switching microglial polarization from M1 to M2, which is likely mediated by APOE/TREM2/NF-κB pathway, and thus ameliorate neurological function

In this study, we basically elucidated the potential mechanism of aOECs transplantation resulting in functional improvement after SCI and the underlying molecular signaling pathway (Fig. 9). Nevertheless, there are still some limitations that need to be improved in our present study. First, we defined M1/M2 based on the classic and common markers (iNOS, CD86, Arg-1 and CD206), rather than a broader analysis of the gene array to define M1/M2 subgroups. Thus it is difficult to exterminate divergent contributions of the intermediate of M1 and M2 phenotypes. Second, TREM2 is not only involved in the NF-κB but also may have impacts on other signaling pathways. In addition, other transcription factors can also participate in regulating NF-κB activity. Third, aOECs release numerous bioactive factors that might have influenced TREM2 function, and it is unknown whether there exists other ligands for TREM2 apart from APOE in aOECs-CM. Additionally, considering the complexity of aOECs-CM component, there might exist other bioactive factors affecting TREM2 function. Anyway, the present finding is of paramount significance to enrich the understanding of the underlying molecular mechanism of aOECs-based therapy for SCI.

## Conclusion

In summary, CCM-treated OECs transplantation can efficiently promote the recovery of neurological function after SCI in rats. The underlying molecular mechanism of the potential therapy is intimately associated with aOECs switching microglia phenotype from M1 to M2 after SCI. In addition, the APOE/TREM2/NF-κB signaling axis is implicated in the mechanism underlying microglial polarization from M1 to M2 phenotype. Therefore, this study is likely to provide a novel therapeutic approach for CCM-activated OECs-based therapy for SCI.

### Electronic Supplementary Material

Below is the link to the electronic supplementary material.

**Supplementary Fig. 1**: Dissection and acquisition of olfactory bulbs and cortices from SD rats. **(a)** Olfactory bulbs. **(b)** Cerebrum of neonatal SD rat. **(c)** Cortices. **Supplementary Fig. 2**: Schematic diagram of spinal cord tissue extracted for western blot. **Supplementary Fig. 3**: Tissue processing and harvesting at the designed time points. **(a)** Perfusion at seven days post-transplantation. **(b)** The spinal cord tissue harvested for preparing frozen sections. **Supplementary Fig. 4**: The activation of OECs after treatment by CCM. **(a)** Quantification of OECs proliferation after CCM treatment at the different time points by CCK-8 assay (n = 3, *P<0.05, **P<0.01, compared with control group). **(b)** Western blot analysis of TG2 and PSR expression level in CMM-treated OECs at 1d, 2d and 3d. **c-d.** Quantification of TG2 and PSR relative expression in OECs under indicated treatments (n = 3, *P<0.05, **P<0.01, ***P<0.01, compared with control group). **Supplementary Table 1**: Primer sequences of siRNAs targeting TREM2.

## Data Availability

The datasets used during the current study are available from the corresponding author on reasonable request.

## Abbreviations

aOECs: CCM-activated olfactory ensheathing
aOECs-CM: aOECs conditional medium
APOE: Apolipoprotein E
BBB: Basso, Beattie, and Bresnahan
CCM: Curcumin
CNS: Central nervous system
EGFP: Enhanced green fluorescent protein
OECs: Olfactory ensheathing cells
OECs-CM: OECs conditional medium
LPS: Lipopolysaccharide
SCI: Spinal cord injury
TREM2: Triggering receptor expressed on myeloid cells 2
