# Supplementary material for: Curcumin-activated Olfactory Ensheathing Cells Improve Functional Recovery After Spinal Cord Injury by Modulating Microglia Polarization Through APOE/TREM2/NF-κB Signaling Pathway
Source: J Neuroimmune Pharmacol. 2023 Sep 2;18(3):476–94. doi: 10.1007/s11481-023-10081-y (PMC10577109; doi:10.1007/s11481-023-10081-y)
Supplement: Supplementary file 1 — Supplementary Fig. 1: Dissection and acquisition of olfactory bulbs and cortices from SD rats. (a) Olfactory bulbs. (b) Cerebrum of neonatal SD rat. (c) Cortices. Supplementary Fig. 2: Schematic diagram of spinal cord tissue extracted for western blot. Supplementary Fig. 3: Tissue processing and harvesting at the designed time points. (a) Perfusion at seven days post-transplantation. (b) The spinal cord tissue harvested for preparing frozen sections. Supplementary Fig. 4: The activation of OECs after treatment by CCM. (a) Quantification of OECs proliferation after CCM treatment at the different time points by CCK-8 assay (n = 3, *P<0.05, **P<0.01, compared with control group). (b) Western blot analysis of TG2 and PSR expression level in CMM-treated OECs at 1d, 2d and 3d. c-d. Quantification of TG2 and PSR relative expression in OECs under indicated treatments (n = 3, *P<0.05, **P<0.01, ***P<0.01, compared with control group). Supplementary Table 1: Primer sequences of siRNAs targeting TREM2. [file 11481_2023_10081_MOESM1_ESM.docx]

**Figure 1** Dissection and acquisition of olfactory bulbs and cortices from SD rats.

**a b c**

**
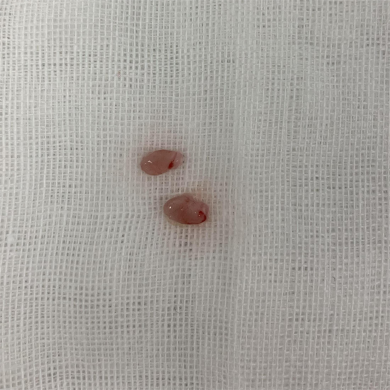

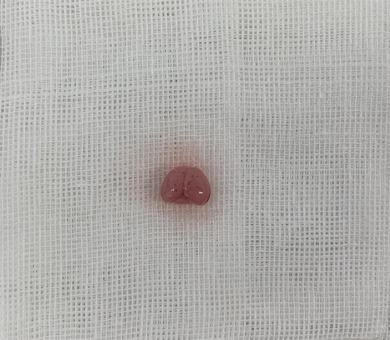

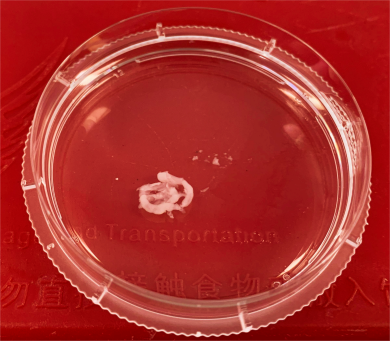
**

**Figure 2** Schematic diagram of spinal cord tissue extracted for western blot.

**
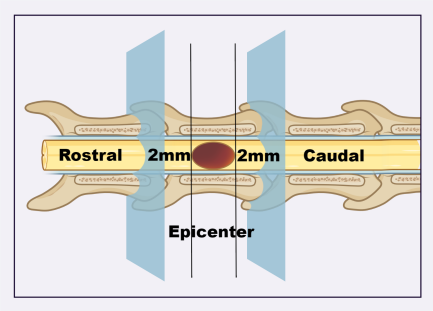
**

**Figure 3** Tissue processing and harvesting at the designed time points.

**a** **b**

**
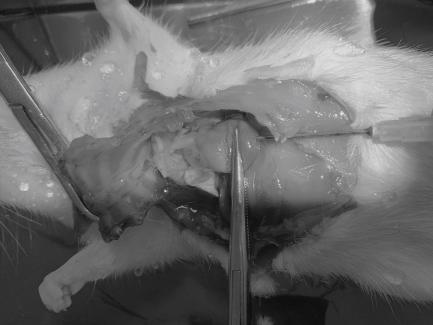
**
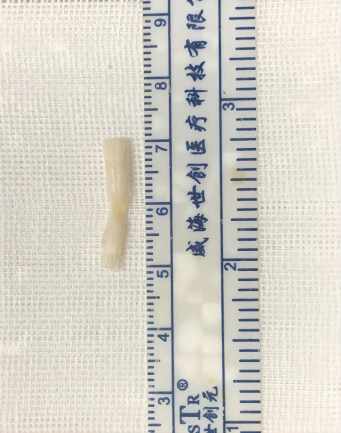


**Figure 4** The activation of OECs after treatment by CCM.


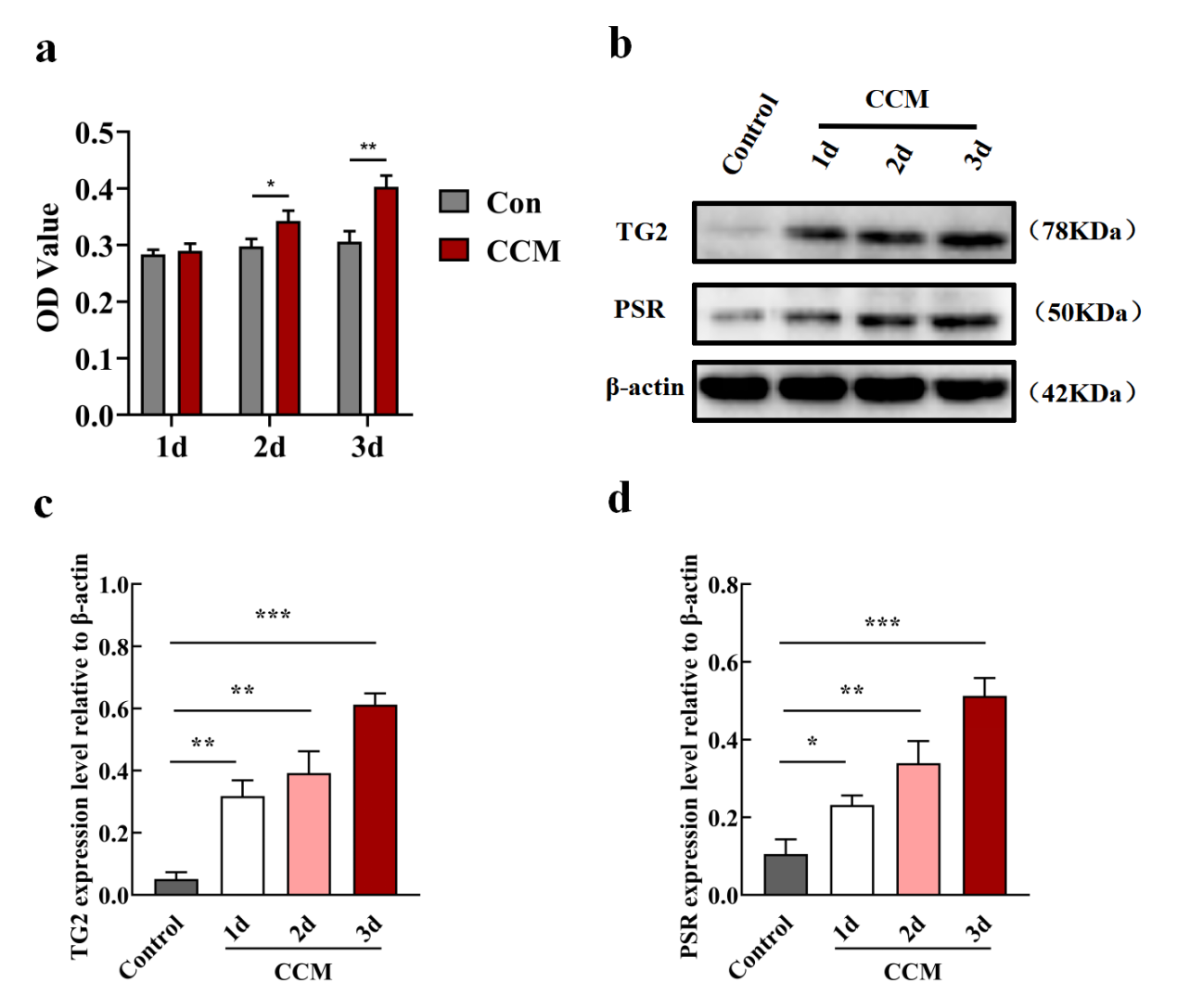


**Table 1** Primer sequences of siRNAs targeting TREM2.

| **Target gene** | **Sense（5'-3'）** | **Anti-sense（5'-3'）** |
| --- | --- | --- |
| TREM2 1 | CCCUCUAGAUGACCAAGAUTT | AUCUUGGUCAUCUAGAGGGTT |
| TREM2 2 | CCUCCUCUUUCCAAGGAAUTT | AUUCCUUGGAAAGAGGAGGTT |
| TREM2 3 | UCUCCUGAGCAAGUUUCUUTT | AAGAAACUUGCUCAGGAGATT |
